# Neuromuscular Fatigue Affects Calf Muscle Activation Strategies, but Not Dynamic Postural Balance Control in Healthy Young Adults

**DOI:** 10.3389/fphys.2022.799565

**Published:** 2022-01-27

**Authors:** Giuseppe Marcolin, Marta Cogliati, Alessandro Cudicio, Francesco Negro, Riccardo Tonin, Claudio Orizio, Antonio Paoli

**Affiliations:** ^1^Department of Biomedical Sciences, University of Padua, Padua, Italy; ^2^Department of Clinical and Experimental Sciences, University of Brescia, Brescia, Italy; ^3^School of Human Movement Sciences, University of Padua, Padua, Italy

**Keywords:** muscle fatigue, high density EMG, dynamic balance, balance control, exercise

## Abstract

Neuromuscular fatigue could negatively affect postural balance, but its effects on dynamic postural regulation are still debated. This study aimed to investigate whether a fatigue protocol on calf muscle could affect muscle activation strategies and dynamic balance performance. Seventeen male adults (age 24.1 ± 4.6 years; height 183.9 ± 7.2 cm; weight 80.2 ± 7.2 kg) volunteered in the study. They performed a dynamic test on an instrumented platform, which provided anterior-posterior oscillations on the sagittal plane, before and after a localized fatigue protocol. High-density surface electromyographical (EMG) signals were recorded bilaterally from the soleus and the medial gastrocnemius muscles. The fatigue protocol, consisting of two quasi-isometric tiptoe standing exercise to failure with a fixed load, did not affect the global dynamic balance performance. Conversely, the frequency value corresponding to 95% of the total power spectrum density of the angular displacement signal increased after fatigue (from 1.03 ± 0.42 to 1.31 ± 0.42 Hz; *p* < 0.05). The EMG analysis showed a significant difference in the PRE/POST fatigue ratio of the root-mean-square (RMS) between the soleus and the gastrocnemius medialis muscles. No differences were detected for the coefficient of variation and the barycenter coordinates of the RMS EMG values between muscles and sides. The variations in the frequency content of the angular displacement and EMG activity across muscles may be related to an increase in the calf muscles stiffness after fatigue. The role of neuromechanical calf muscle properties seems to be relevant in maintaining the dynamic postural performance after a quasi-isometric fatigue protocol until failure.

## Introduction

Postural balance control is fundamental both in everyday life and sports activities. If posture represents the position of the different body segments (Paillard, 2017), balance is defined as the capacity to keep the center of pressure (COP) within the base of support to avoid falling (Winter et al., 1996). The scientific literature differentiates static from dynamic postural balance control: the first is described as the ability of a person to maintain the balance in an unperturbed environment (e.g., quiet standing on a firm surface) (Macpherson and Horak, 2013); the second represents the ability of a person to cope with sudden changes of postural conditions (e.g., displacement of the base of support) or external mechanical perturbations (e.g., forces applied to large body segments) (Paillard, 2019). An efficient postural balance control can reduce the fall risk and contribute to the efficiency of motor/sports performance (Paillard, 2017). On this topic, muscle fatigue has been demonstrated to affect neuromuscular control negatively, and thus postural balance, since fatigue can alter the somatosensory inputs (e.g., the threshold of muscle spindle discharge) (Gribble and Hertel, 2004), motor neuron discharge statistics (Enoka, 2012) and muscle stiffness (Granata et al., 2004). The effect of fatigue on postural balance has been investigated both after a total body exercise as uphill walking, cycling, or running (Nardone et al., 1997, 1998) and following exercises involving single joints (i.e., ankle, knee, or hip joints) (Gribble and Hertel, 2004; Salavati et al., 2007; Gimmon et al., 2011). Indeed, it has been shown that strenuous treadmill exercise negatively affects the COP sway measured on a force platform during quiet standing and that the effect vanishes within a few minutes (Nardone et al., 1998). Moreover, COP sway is little influenced when exercises are executed below the anaerobic threshold (Nardone et al., 1997). Conversely, COP is primarily affected when exercises envisage strong intensity muscle contractions providing a significant amount of proprioceptive stimulation (e.g., running vs. cycling) (Nardone et al., 1997; Zemková, 2014). In addition to this, localized muscle fatigue seems to significantly influence postural balance control when proximal lower limb muscle groups are involved (Gribble and Hertel, 2004; Salavati et al., 2007). Furthermore, plantar flexor muscles are crucial for maintaining the upright posture (Loram et al., 2005) due to the anterior position of the COP concerning the ankle joints (Winter, 1995). Indeed, when ankle plantar flexors are fatigued, the postural balance impairment during static posturography mainly occurs in the sagittal plane (Gimmon et al., 2011). To date, only a few studies reported at the same time the effect of fatigue both on postural balance performance and plantar flexor muscle activity (Ritzmann et al., 2016; Watanabe et al., 2018). In particular, the decrement of static balance performance after fatigue has been explained by a change of the neuromuscular control that caused an increase of muscle co-contractions and, as a consequence, of joint stiffness (Ritzmann et al., 2016). Watanabe et al. (2018) demonstrated with bipolar surface electromyography that the worsening of balance performance was accompanied by a reduction of the low-frequency common input to the plantar flexor muscles.

Although it seems conceivable to perform dynamic besides static tests because they provide more discriminant information on postural balance performance (Petró et al., 2017), the literature on the effect of fatigue on dynamic postural control is still scarce. Miller and Bird (1976) have shown that localized fatigue only on some specific muscle groups can affect the dynamic postural control performance. More recent studies highlighted contradicting results on the effect fatigue has on dynamic balance control (Salavati et al., 2007; Marcolin et al., 2016). Regardless of the results, these studies demonstrate that compensatory mechanisms may occur at the neural or peripheral levels of specific muscle groups to overcome the adverse effects of muscle fatigue on dynamic postural control.

Nevertheless, none of the previous studies investigated the electromyographical (EMG) activity of the muscles fatigued while executing the dynamic balance tests.

Therefore, the present study aimed to increase the body of knowledge on the interplay between neural and mechanical components of the dynamic postural balance control in healthy subjects following a standardized fatigue protocol designed to involve the ankle dorsiflexor muscles. Specifically, we aimed to investigate if the fatigue protocol could influence the calf muscle activation strategies during the performance of a dynamic postural balance test. We hypothesized that compensation mechanisms in the calf muscle activation strategies would be engaged to minimize the effects of the fatigue protocol on the dynamic postural balance performance.

## Materials and Methods

### Participants

A total of 17 young male adults (age 24.1 ± 4.6 years; height 183.9 ± 7.2 cm; weight 80.2 ± 7.2 kg) participated in the study. Exclusion criteria were the presence of pathologies to the vestibular system, the presence of non-corrected visual refractive errors, and the presence of acute or chronic pathologies affecting muscles, tendons, and joints of the lower limbs. Each participant was instructed on the experimental protocol before giving his written informed consent to the participation. The study was approved by the ethical committee of the Department of Biomedical Sciences, University of Padova.

### Experimental Procedure

#### Dynamic Postural Balance

**FIGURE 1:**
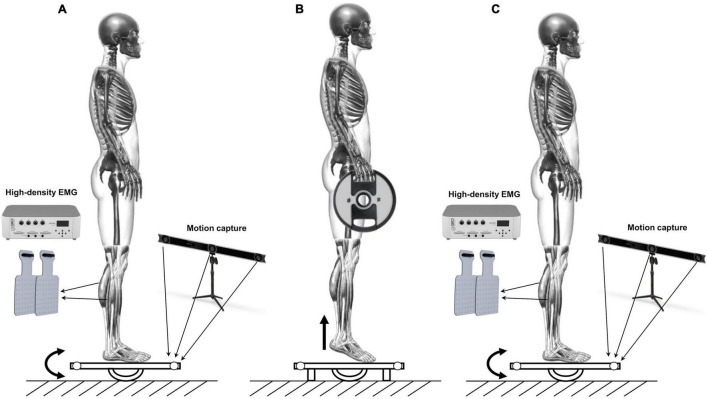
Schematic representation of the experimental protocol. **(A)** 40 s trial on the oscillating platform. **(B)** Standing calf to failure gripping a 10 kg disk on each hand. **(C)** 40 s trial on the oscillating platform after the fatigue protocol.

The dynamic postural balance of the participants was assessed barefoot on a custom-made marine plywood platform (length: 50 cm; width: 50 cm; height: 8.5 cm) which rotated along a single axis providing anterior-posterior perturbations on the sagittal plane. A marine plywood semicylinder allows the platform to rotate 16 deg, both anteriorly and posteriorly. The surface of the platform was covered with an anti-slip film. Measurements were taken before and after a fatigue protocol. More in detail, each participant had to maintain an upright posture with arms along their sides. The midpoint of each foot, identified as half the distance between the medial malleolus and the head of the first metatarsal head, was marked and positioned over the line which divided into two equal parts the surface of the platform. Feet had to be kept parallel with the step width equal to the pelvis width. The protocol consisted of two trials of 40 s with 3-min rest in between. During the trials, participants were asked to maintain the platform parallel to the ground while straight gazing at a target positioned in front of them at 2.5 meters. After that, the fatigue protocol occurred. It consisted of two quasi-isometric tiptoe standing calf exercises to failure gripping a 10-kg disk on each hand. The participants had to stand on their tiptoes until failure: the first standing calf ended when the heels touched the platform, which had been preventively locked, avoiding oscillations. After recovery of 1 min, participants performed the second quasi-isometric standing calf. The time duration of each quasi-isometric standing calf to failure was recorded. Indeed, inducing an incapacity to continue a particular effort has been described as one of the three techniques generating fatigue (Paillard, 2012). During the fatigue protocol, the operators verbally encouraged participants to do their best. Immediately after the participants’ heels touched the platform in the second standing calf to failure, the platform was unlocked, and participants performed again a 40-s trial with the same indications followed in the two previous trials. The representation of the experimental setup is shown in Figure 1.

The anterior and posterior oscillations of the platform were recorded by an optoelectronic system (V120 Trio, OptiTrack, United States) that collected at a sampling frequency of 120 Hz the three-dimensional position of two reflective markers applied on the edge of the platform.

#### High-Density Surface Electromyography

High-density surface EMG signals were recorded from the soleus and the medial gastrocnemius muscles of each leg. The recordings were made in monopolar derivation with two-dimensional adhesive grids (Spes Medica, Salerno, Italy) of 13 (rows) × 5 [columns electrodes 8/8/spaced by 8 mm (1 mm diameter)] on each muscle. The high-density surface EMG signals were acquired using an EMG-USB2 + amplifier (256 channels plus 16 auxiliary channels; OT Bioelettronica, Turin, Italy). After the skin was shaved and cleaned with abrasive paste and water, the electrode grids were covered with a double-sided foam and the electrode cavities were filled with conductive paste (Spes Medica, Salerno, Italy). The matrices were positioned following the guidelines according to Barbero et al. (2012). The surface EMG signals were amplified (EMG-USB2+, OT Bioelectronics, Italy), band-pass filtered (−3 dB, from 20 to 500 Hz), and sampled at 2,048 Hz with the OT Biolab software (OT Bioelettronica, Turin, Italy). Surface EMG signals were amplified with variable gain (500–2,000 V/V). Although the tibialis anterior was not involved during the fatigue protocol, bipolar surface EMG signal was recorded by two self-adhesive pre-gelled electrodes (diameter 1 cm), placed at an interelectrode distance of 20 mm as control, allowing to exclude the instauration of compensatory activation patterns. The bipolar EMG signals were filtered with a 512 Hz low pass filter before being recorded. The bipolar surface EMG signals were collected by a16 channel surface electromyographic signal amplifier (OT Bioelettronica, Turin, Italy).

Data were analyzed offline using MATLAB 2019b (The MathWorks, Inc., Natick, MA, United States). The kinematic and electromyographic data were collected synchronously through a manual trigger.

### Data Analysis

#### Kinematic Data Analysis

The platform’s oscillation was calculated with a custom SmartAnalyzer program (BTS Bioengineering, Milan, Italy) starting from the three-dimensional coordinates of the two reflective markers. Thus, 0°corresponded to the platform parallel to the ground, positive angular values to posterior oscillation (i.e., ankle dorsiflexion), and negative angular values to anterior oscillation (i.e., ankle plantarflexion). The platform’s whole range of motion was 32° (16° anteriorly and 16° posteriorly, respectively). Three parameters were considered for the assessment of the dynamic postural balance similar to a previous work (Sarto et al., 2020): the overall integral of the time-angle curve (Full Balance, FB); the time participants kept the platform between 4° of anterior and 4° of posterior oscillation (Fine balance, FiB); the time participants kept the platform between 8° of anterior and 8° of posterior oscillation (Gross balance, GB). Moreover, we calculated the power spectral density (PSD) as the square of the amplitude spectrum estimated by fast Fourier transformation of the time-angle input signal. PSD allowed highlighting which oscillation frequencies were predominant in the signal input, providing information not clearly visible in the time domain. Specifically, we adopted the in-built MATLAB periodogram function with a rectangular window of duration equal to the signal length (The MathWorks, Inc., MA, United States). Then, the integral of the PSD curve was calculated to extrapolate for each trial the frequency values corresponding to the 50% (PSD_50%) and 95% (PSD_95%) of the total PSD.

#### Electromyographical Data Analysis

The root mean square map amplitude (RMS_MAP) of the EMG signals was calculated during the dynamic tasks performed on the platform before and after the fatigue protocol. RMS_MAP was estimated from the average of all differential channels of the electrode grid (Martinez-Valdes et al., 2020), as this provides more reliable estimates of muscle activity (Vieira and Botter, 2021). The RMS_MAP values calculated on the entire high-density surface grid for each calf muscle were averaged over the 40-s signal duration. Additionally, the root mean square amplitude (RMS) derived from a bipolar derivation with larger electrodes was calculated. Specifically, the monopolar EMG signals from two sets of five electrodes in close proximity were averaged to derive the EMG signals detected from two large electrodes. The derived EMG signals were subtracted to estimate a bipolar EMG derivation with an interelectrode distance of 1.6 cm, similar to a previous study (Del Vecchio et al., 2017). The RMS values for each large bipolar configuration were extracted for each calf muscle and averaged over the 40-s signal duration. The RMS values were also calculated for the tibialis anterior bipolar EMG signals (RMS_TA) in the same segments. In our experimental protocol, we could not measure the maximal voluntary activation of the investigated muscles. Therefore, the variation of the RMS_MAP, RMS and RMS_TA values before and after the fatigue protocol (PRE/POST*100) was compared across muscles and sides. Additionally, the spatial distribution of the muscle activity was estimated by extracting the barycenter coordinates (BAR_X and BAR_Y, respectively) and the coefficient of variation (CoV) of the RMS maps for the soleus and the medial gastrocnemius muscles on both monopolar and differential signals. In order to match with the RMS analysis, both barycenter coordinates and CoV variables were normalized to the PRE condition.

### Statistical Analysis

A two-tailed paired *t*-test was carried out to compare the kinematic parameters before and after the fatigue protocol and compare the duration of the two fatigue trials. This analysis was performed with GraphPad Prism version 4.00 (GraphPad Software, San Diego, CA, United States). Cohen’s *d* effect size (*d*) was calculated with G*Power 3.1 (Faul et al., 2007) and was interpreted as follow: 0.00–0.19: trivial; 0.20–0.59: small; 0.60–1.19: moderate; 1.20–1.99: large and >2.00: very large (Hopkins et al., 2009). A two-way ANOVA for repeated measures was employed to compare the ratios of the EMG variables (RMS, RMS_MAP, COV, BAR_X, and BAR_Y) with factors of side (right and left) and muscle (soleus and gastrocnemius muscle). Additionally, a paired *t*-test was performed for both muscles to compare the EMG activity before and after the fatigue protocol. To compare the RMS_TA percentage values, a One-Sample *t*-test was performed for left and right legs independently. The significant level was set at *p* < 0.05 for all the statistical tests performed. All EMG statistical analyses were performed with IBM SPSS Statistics 26 (IBM, Armonk, NY, United States).

## Results

**FIGURE 2:**
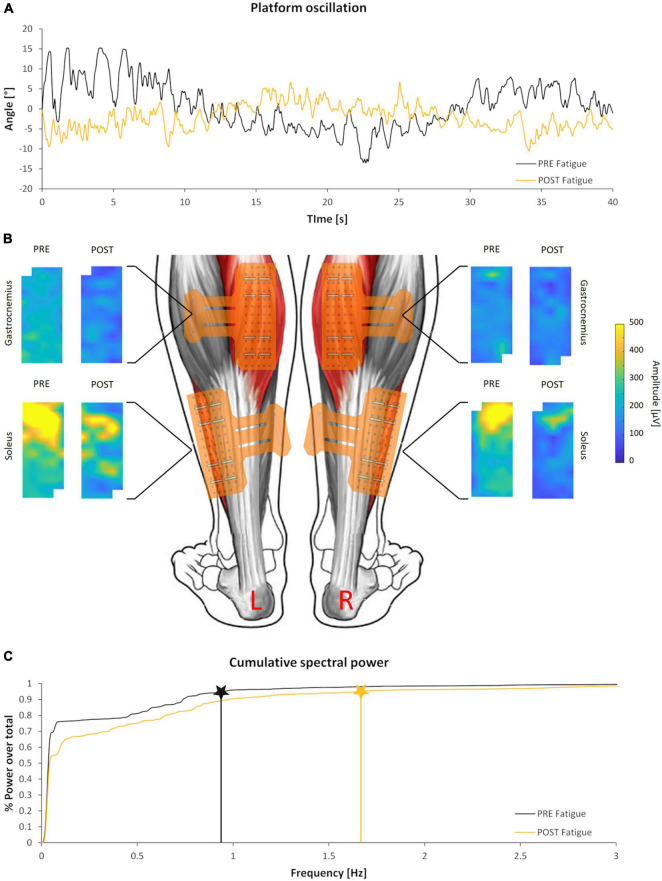
Kinematics and electromyographical (EMG) outcomes of a representative subject. **(A)** Angular displacement signal of the oscillating platform before (black) and after (yellow) the fatigue protocol. **(B)** Colored maps representing the spatial distribution of EMG activity before and after the fatigue protocol of the calf muscles analyzed. Maps are scaled to the same amplitude to highlight the differences between and within muscles. **(C)** Integral of the PSD time-angle curve and correspondent PSD_95% values before (black) and after (yellow) the fatigue protocol.

The time duration of the second fatigue trial (196.7 ± 80.10 s) was shorter than the first one (279.8 ± 97.49 s) and the difference was statistically significant (*p* < 0.01; *d* = 0.92), highlighting the effectiveness of the fatigue protocol adopted. Nonetheless, the fatigue protocol did not affect the dynamic balance performance on the oscillating platform. In fact, for both FB, FiB, and GB, no statistically significant worsening or improvement was detected. Conversely, the frequency value corresponding to 95% of the total PSD (PSD_95%) showed a statistically significant increment (*p* < 0.05; *d* = 0.67), underlying a shift toward higher frequencies of oscillation in the dynamic postural balance test after the fatigue protocol. An example of the angular displacement signal before and after the fatigue protocol is reported in Figure 2A together with the integral of the PSD time-angle curve (Figure 2C). Numerical data of both kinematics and power spectral density results are presented in Table 1.

**TABLE 1:** Kinematics and power spectral density results before and after the fatigue protocol.

	*PRE*	*POST*	*p*-value	Cohen’s *d*
Full balance (FB)	133.5 ± 44.16	134.3 ± 24.58	0.94	0.02
Gross balance (GB) [s]	36.78 ± 3.06	38.19 ± 1.69	0.08	0.53
Fine balance (FiB) [s]	27.92 ± 6.49	25.80 ± 4.40	0.22	0.37
PSD_50% [Hz]	0.15 ± 0.17	0.13 ± 0.10	0.66	0.16
PSD_95% [Hz]	1.03 ± 0.42	1.31 ± 0.42	0.02	0.67

*Data are expressed as means and standard deviations.*

**FIGURE 3:**
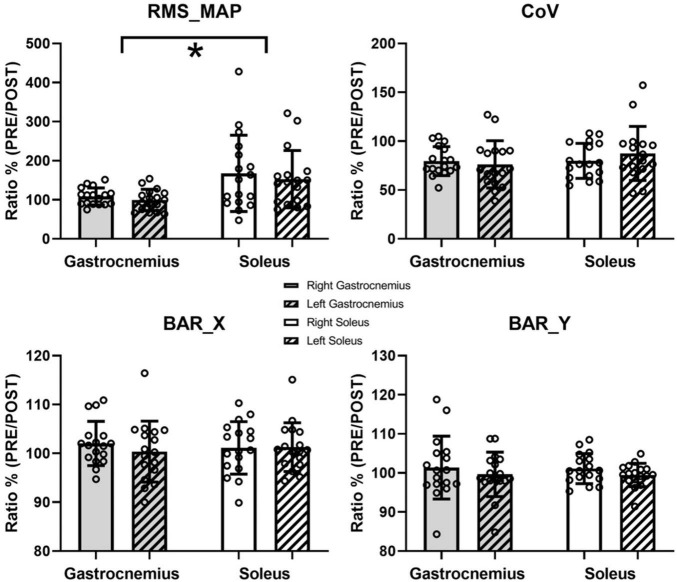
Results of the EMG analysis. Data are presented as means and standard deviations. RMS_MAP, CoV, and barycenter coordinate (BAR_X and BAR_Y) are reported. * represents statistically significant differences between muscles (*p* < 0.05).

Figure 2B shows the unnormalized RMS activity maps of each muscle and side before and after the fatigue protocol for a representative subject. Previous studies have well described the representation through color maps that characterize the electromyographic activity (Orizio et al., 2017; Falla and Gallina, 2020; Pradhan et al., 2020). The parameters extracted from the EMG analysis showed significant differences after the fatigue protocol. In particular, the normalized RMS_MAP values averaged over the full matrices for all subjects demonstrated an effect of muscle (*F* = 7.4, *p* = 0.015, ηp2 = 0.31) but not of side (*F* = 3.4, *p* = 0.083, ηp2 = 0.176, Figure 3). Similarly, results of the RMS values calculated from the large bipolar derived from the surface EMG matrices showed effect of muscle (*F* = 8.4, *p* = 0.013, ηp2 = 0.345) but no effect of side (*F* = 2.7, *p* = 0.116, ηp2 = 0.148), demonstrating a larger variation of normalized RMS values in the soleus muscle during the dynamic activity after the fatigue protocol. Notably, EMG activity showed a statistically significant reduction for the soleus (left side: *p* = 0.028, *d* = 0.52; right side: *p* = 0.022, *d* = 0.68) and no variation for the gastrocnemius medialis (left side, *p* = 0.496; right side, *p* = 0.440) after the fatigue protocol.

Conversely, the spatial distribution of the RMS maps over the muscles during the dynamic activity did not change after the fatigue protocol. Specifically, the PRE/POST variations in the spatial variability (CoV for RMS values) did not show statistical difference, with no muscle (*F* = 1.163, *p* = 0.297, ηp2 = 0.068) or side (*F* = 0.306, *p* = 0.588, ηp2 = 0.019) effects (Figure 3). Neither the ratios of the barycentres of the RMS activity showed statistically significant effects considering BAR_X (muscle, *F* = 0, *p* = 0.994, ηp2 = 0; side, *F* = 0.414, *p* = 0.529, ηp2 = 0.025) and BAR_Y (muscle, *F* = 0.06, *p* = 0.81, ηp2 = 0.04; side, *F* = 1.031, *p* = 0.325, ηp2 = 0.061). No changes were found between the right (*p* = 0.115) and left (*p* = 0.239) tibialis anterior for the normalized RMS_TA values after the fatigue protocol.

## Discussion

The present study aimed to investigate the effect of a neuromuscular fatigue protocol on dynamic postural performance and calf muscle activation strategies. The main finding of the current study was that the reduction of the EMG activity of the soleus during the dynamic balance task after fatigue did not affect the global dynamic postural balance performance. We can speculate this behavior is part of calf muscle compensatory mechanisms acting to minimize the effect of fatigue on the dynamic balance performance.

Previous studies showed that changes in the dynamic postural balance performance depend on the characteristics of the fatigue protocol. For example, localized muscle fatigue has been shown to affect dynamic balance only when knee and hip flex-extensors, but not plantar flexors, were fatigued (Miller and Bird, 1976). Similarly, it has been demonstrated that exercise inducing fatigue in plantar flexors had no effects on postural control (Adlerton and Moritz, 1996; Caron, 2004; Rojhani-Shirazi et al., 2019). In particular, Alderton and colleagues suggested that compensatory mechanisms are put in place to counteract plantar flexors fatigue (i.e., increased reflex activity in muscle spindles or increased muscle stiffness due to fatigue) and maintain an unaltered postural control. Nonetheless, we are still far away from a clear consensus since in other similar studies, localized muscle fatigue of the plantar flexors has been demonstrated to decrease both static (Yaggie and McGregor, 2002; Vuillerme et al., 2006; Gimmon et al., 2011) and dynamic (Gribble and Hertel, 2004; Salavati et al., 2007) postural control. To this extent, our findings support the body of literature that found no differences in the dynamic balance performance after a localized plantar flexors fatigue.

Interestingly, when generalized muscle fatigue was induced, the dynamic postural behavior was similar to the one reported in the current study. In line with this, ultramarathon runners fatigued by more than 15-h race (Marcolin et al., 2016) or recreational runners after 25-min treadmill running (Marcolin et al., 2019) showed a similar dynamic balance performance before and after fatigue. Therefore, although it appears to exist causation between muscle fatigue and postural control, this relation seems highly dependent on the type of the fatigue protocol (i.e., localized on specific muscles or generalized) and postural task (i.e., static or dynamic). The reasons for such discrepancies may be found in the influence of fatigue on the interaction between neural and mechanical components of the human postural control. Alternatively, we can speculate that an increase in the vigilance level after the fatiguing could occur (Paillard, 2012), improving the effectiveness of the descending drive for the postural muscle motor neurons activation and the integration of afferent information (Nardone et al., 1998).

Nonetheless, the first hypothesis seems the most robust since muscle fatigue is well recognized reducing the rate of force development (Allen et al., 2008), altering proprioceptive information from muscle afferents (Vuillerme and Boisgontier, 2008), inducing metabolic inhibition of the contractile process and excitation-contraction coupling failure, and increasing joint stiffness (Gajdosik, 2001; Zhang and Zev Rymer, 2001; Hoang et al., 2007).

Our findings observed an increase in the high-frequency content (i.e., an increment of PSD_95%) of the angular displacement signal (Figure 2C and Table 1) after the fatigue protocol. The frequency content change could have been indirectly related to a higher rigidity of the triceps surae muscle group that emphasized this aspect of the motor control mechanical outcome. Indeed, Baratta and Solomonow (1992) reported that the more rigid the system, the less the tension dynamics filter by its viscoelastic elements. Stiffness could also be reflected in a more effective force transmission (Bojsen-Møller et al., 2005; Cogliati et al., 2020). Moreover, previous studies suggested that increased joint stiffness (Winter et al., 1998; Caron, 2003; Corbeil et al., 2003) plays a determinant role in postural control management. Although recent researches underlined limitations in this interpretation for static postural control (Morasso and Schieppati, 1999; Loram et al., 2005, 2009), it is reasonable to assume a key role of muscle stiffness during dynamic postural tasks (Adlerton and Moritz, 1996), like the one performed in the current study. Future studies are warranted to investigate the possible relationship between the frequency components of the kinematic signal detected in our experiment and the motor unit neural control features retrievable by high-density EMG processing (Negro et al., 2009). According to the theory that increased stiffness of plantar flexors could counterbalance the effect of fatigue letting unaltered the dynamic balance performance on the platform, it was not surprising to observe a difference in the variation of the surface EMG amplitude between soleus and gastrocnemius muscles after the fatigue protocol (Figure 3). The unchanged EMG activity of gastrocnemius medialis may provide evidence of higher soleus muscle engagement after the fatigue protocol performing a dynamic balance test with a relatively small range of motion. Interestingly, we did not observe changes in the coefficient of variation and the barycenter coordinates of the RMS values across the EMG matrices, highlighting no modifications in the spatial activation strategies of the calf muscles performing the dynamic balance task after fatigue. In line with our work, previous studies have found minimal variations of the RMS of the barycenter during isometric or dynamic fatigue tasks in healthy individuals (Falla and Farina, 2007; Arvanitidis et al., 2021). This finding certainly deserves further investigation.

Last, it is relevant to mention a limitation of the present work. Although participants were instructed to maintain an upright posture with arms along their sides, we observed slight movements of the trunk relative to the pelvis that we did not control to ensure the participants performing the task at their best. Indeed, those tiny motions could have somehow affected the participants’ center of mass location and consequently could have marginally influenced the calf muscle activation.

## Conclusion

The present study hypothesized compensatory mechanisms in the triceps surae muscles before and after the fatigue protocol that allowed maintaining similar performance levels in the dynamic postural task on the oscillating platform. Specifically, we highlighted two compensation strategies consequent the fatigue protocol: (i) a difference between soleus and gastrocnemius activity PRE/POST fatigue; (ii) an expansion of the frequency content of the platform oscillations, likely due to an overall increase of the calf muscles stiffness. Although we hypothesized causation between muscle fatigue and neuromechanical compensation strategies, further studies need to deepen these compensatory mechanisms investigating the key role of muscle stiffness, the neuromuscular complexity of the physiological parameters through fluctuation and variability analysis (e.g., approximate entropy and detrended fluctuation analysis), and the neural control features of motor units retrievable by high-density EMG processing.

## Data Availability Statement

The raw data supporting the conclusions of this article will be made available by the authors, without undue reservation.

## Ethics Statement

The studies involving human participants were reviewed and approved by the Ethical Committee of the Department of Biomedical Sciences, University of Padua. The patients/participants provided their written informed consent to participate in this study.

## Author Contributions

GM, MC, CO, and AP conceived and designed the experiments. GM, MC, AC, FN, and RT performed the experiments. GM, MC, AC, FN, CO, and RT analyzed the data. AP and CO contributed to the materials. GM, MC, AP, and FN wrote the manuscript. All authors approved the final version of the manuscript.

## Conflict of Interest

The authors declare that the research was conducted in the absence of any commercial or financial relationships that could be construed as a potential conflict of interest.

## Publisher’s Note

All claims expressed in this article are solely those of the authors and do not necessarily represent those of their affiliated organizations, or those of the publisher, the editors and the reviewers. Any product that may be evaluated in this article, or claim that may be made by its manufacturer, is not guaranteed or endorsed by the publisher.
